# Kisspeptin, Neurokinin B, and Dynorphin Expression during Pubertal Development in Female Sheep

**DOI:** 10.3390/biology10100988

**Published:** 2021-09-30

**Authors:** Eliana G. Aerts, KaLynn Harlow, Max J. Griesgraber, Elizabeth C. Bowdridge, Steven L. Hardy, Casey C Nestor, Stanley M. Hileman

**Affiliations:** 1 Department of Physiology and Pharmacology, West Virginia University, P.O. Box 9229, Morgantown, WV 26506, USA; ea0072@mix.wvu.edu (E.G.A.); mjg0054@mix.wvu.edu (M.J.G.); ebowdrid@hsc.wvu.edu (E.C.B.); shardy@hsc.wvu.edu (S.L.H.); 2 Department of Animal Science, North Carolina State University, Raleigh, NC 27695, USA; keharlow@ncsu.edu (K.H.); ccnestor@ncsu.edu (C.C.N.); 3 Department of Neuroscience, West Virginia University, Morgantown, WV 26506, USA

**Keywords:** kisspeptin, neurokinin B, dynorphin, puberty, sheep, LH

## Abstract

**Simple Summary:**

Mistiming of puberty onset has negative consequences for humans and livestock. Puberty depends on increased brain secretion of gonadotrophin-releasing hormone (GnRH), which causes pituitary luteinizing hormone (LH) release. Luteinizing hormone then stimulates estrogen release from the ovary and, eventually, the first ovulation. The brain neural systems that control the pubertal increase in GnRH and LH are not completely known. Neurons co-expressing kisspeptin, neurokinin B (NKB), and dynorphin (called KNDy neurons) are critical for puberty, with kisspeptin and NKB stimulating and dynorphin inhibiting LH secretion. Herein, we used female sheep at prepubertal, peripubertal, and postpubertal ages and hypothesized that kisspeptin and NKB would increase, and dynorphin decrease, during the pubertal increase in LH secretion. We observed that kisspeptin and NKB protein and mRNA expression were evident well before the pubertal increase in LH secretion and did not change with age. We saw no change in numbers of dynorphin neurons, but actually saw an increase in the amount of mRNA per neuron at a postpubertal age. We conclude that puberty-associated increases in GnRH and LH secretion occur without significant changes in KNDy peptide expression and suggest that, while critical, KNDy neurons await inputs from other neural systems to trigger puberty onset.

**Abstract:**

The neural mechanisms underlying increases in gonadotropin-releasing hormone (GnRH) and luteinizing hormone (LH) secretion that drive puberty onset are unknown. Neurons coexpressing kisspeptin, neurokinin B (NKB), and dynorphin, i.e., KNDy neurons, are important as kisspeptin and NKB are stimulatory, and dynorphin inhibitory, to GnRH secretion. Given this, we hypothesized that kisspeptin and NKB expression would increase, but that dynorphin expression would decrease, with puberty. We collected blood and hypothalamic tissue from ovariectomized lambs implanted with estradiol at five, six, seven, eight (puberty), and ten months of age. Mean LH values and LH pulse frequency were the lowest at five to seven months, intermediate at eight months, and highest at ten months. Kisspeptin and NKB immunopositive cell numbers did not change with age. Numbers of cells expressing mRNA for kisspeptin, NKB, or dynorphin were similar at five, eight, and ten months of age. Age did not affect mRNA expression per cell for kisspeptin or NKB, but dynorphin mRNA expression per cell was elevated at ten months versus five months. Thus, neither KNDy protein nor mRNA expression changed in a predictable manner during pubertal development. These data raise the possibility that KNDy neurons, while critical, may await other inputs for the initiation of puberty.

## 1. Introduction

**Figure 1:**
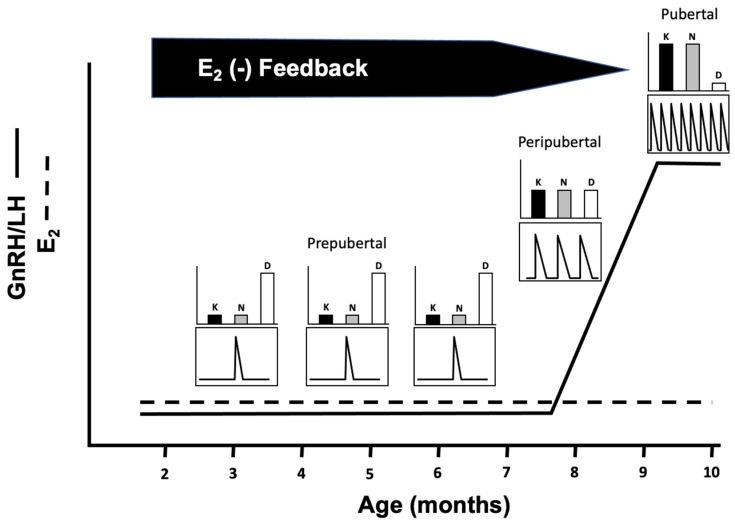
Diagram of changes in mean GnRH/LH secretion (solid line) and LH pulse frequency (lower panel insets) using a model wherein female sheep are OVX and implanted with a constant-release implant of E_2_, indicated by the unchanging levels of E_2_ with development (dotted line). During the prepubertal period (up to ~7 months), sensitivity to E_2_-negative feedback is high and mean GnRH/LH secretion and LH pulse frequency is low. As animals grow and mature, sensitivity to E_2_-negative feedback lessens, leading to an increase in GnRH/LH secretion and LH pulse frequency (~8 months). By ~9 months of age, the normal time of puberty, E_2_-negative feedback has further lessened and GnRH/LH secretion and LH pulse frequency is maximal. The upper panel insets depict our hypothesis regarding changes in the KNDy neuron peptides kisspeptin (K), neurokinin B (N) and dynorphin (D). As kisspeptin and neurokinin B are stimulatory and dynorphin is inhibitory to GnRH/LH secretion, we hypothesize that expression of kisspeptin and neurokinin B will be minimal and that of dynorphin elevated during the prepubertal period in association with reduced GnRH/LH secretion. As the animal grows and matures, expression of the peptides will change in association with increased GnRH/LH secretion such that the expression of kisspeptin and neurokinin B will predominate and dynorphin will recede.

Puberty is a complex process that is critical for the perpetuation of a species and its appropriate timing is important. In humans, both precocious and delayed puberty can have negative health outcomes, which include increased risk of eating disorders, obesity, osteoporosis, cardiovascular disease, breast cancer, depression, substance abuse, and suicide [1,2,3,4,5]. In domestic livestock, delayed puberty can reduce the lifetime productivity of an individual and thereby reduce profitability [6,7,8]. Puberty in mammalian species relies on an increase in the frequency of gonadotropin-releasing hormone (GnRH) pulses, which, in turn, elicits a corresponding increase in luteinizing hormone (LH) pulses. As GnRH cannot be measured in the peripheral circulation, LH makes a useful surrogate as a measure of GnRH release. These greater circulating concentrations of LH drive ovarian follicular development and increase follicular 17β-estradiol (E_2_) production, ultimately leading to a GnRH/LH surge and the first ovulation. Importantly, the prepubertal hiatus in GnRH/LH release is not due to a developmental deficiency in GnRH synthesis or pituitary gonadotrope responsiveness, but instead appears to be due to a central imposition of inhibition or a lack of stimulation, or both [9]. In several species, including sheep, relatively low GnRH/LH release prior to puberty is due to heightened hypothalamic sensitivity to E_2_-negative feedback [9]. This can be clearly shown using an experimental model wherein the ovaries have been removed and replaced with constant-release E_2_ implants [10] (Figure 1). Herein, sensitivity to E_2_-negative feedback is high prepubertally (in sheep at 2–7 months) and GnRH/LH pulse frequency and mean LH levels are very low. As animals age, grow, and mature, they enter a peripubertal period wherein GnRH/LH pulse frequency and mean LH begins to increase (~8 months of age in sheep). In this model, this happens in the face of unchanging circulating E_2_ concentrations, indicating a decreased ability of E_2_ to inhibit GnRH/LH secretion, a phenomenon originally described in rats as the gonadostat hypothesis [11]. As the animal continues to grow and age further, pulsatile GnRH/LH secretion continues to increase, reaching sustained maximal levels, indicative of a severe reduction in the ability of E_2_ to inhibit their secretion (~9 months in sheep). This change in LH secretion has been termed “neuroendocrine puberty” [9] and occurs over a time period that mimics the onset of estrous cycles in ovary-intact female sheep [10]. In primates, while the prepubertal suppression of LH secretion prior to menarche is steroid-independent [12], E_2_-negative feedback does play a role in the period between initial menarche and the establishment of consistent menstrual cycles [13].

Given that GnRH neurons do not express estrogen receptor alpha (ERa) [14,15], the ER isoform necessary for E_2_-negative feedback [16], E_2_ must regulate the activity of GnRH neurons through other E_2_-receptive afferent neurons. Located in the arcuate nucleus (ARC) of the hypothalamus, neurons that coexpress kisspeptin, neurokinin B (NKB), and dynorphin, termed KNDy neurons [17], highly express ERa [18,19] and approximately 60% of GnRH neurons receive synaptic input from KNDy neurons [20]. Kisspeptin has been shown in humans and rodents to be critical for puberty onset as a lack of the peptide or its receptor leads to hypogonadotropic hypogonadism [21,22,23,24]. Given that the vast majority of GnRH neurons express the receptor for kisspeptin, Kiss1R [25,26], and that kisspeptin has been shown to stimulate LH in all species to date [27,28,29,30,31,32,33], kisspeptin is believed to directly stimulate GnRH neurons. Likewise, NKB has been shown to be necessary for puberty onset in humans [34,35], though deletion of NKB in mice is much less impactful [36,37,38]. Given that the receptor for NKB, NK3R, is expressed in ARC kisspeptin neurons, but not GnRH neurons [39], the stimulatory effect of NKB on GnRH/LH secretion [40] is thought to be indirect via activation of KNDy neurons [41]. The role of dynorphin is less explored, but our earlier work in prepubertal female sheep suggests that it may comprise part of the “brake” that suppresses LH secretion prior to puberty [42]. KNDy neurons have been suggested to be the GnRH pulse generator [43,44,45] with NKB acting locally to initiate the pulse by acting on other KNDy neurons, thereby triggering release of kisspeptin, while dynorphin inhibits KNDy neurons to terminate kisspeptin release.

In female sheep, there is only limited data regarding changes in KNDy peptides with puberty. Our previous work showed that numbers of kisspeptin immunopositive cells were greater in ovary-intact ewes in the early part of the follicular phase than at a prepubertal age [40]. However, another study showed that numbers of kisspeptin mRNA expressing cells were similar in ovariectomized (OVX) female sheep-implanted sc with E_2_ at prepubertal, peripubertal, and postpubertal ages [46]. One possible reason for the seeming discrepancy between studies is the reliance on immunocytochemical assessment of kisspeptin protein in one and assessment of kisspeptin mRNA by in situ hybridization in the other. In this study, we evaluated both mRNA and protein expression for kisspeptin within the same animals. In addition, we also evaluated mRNA and protein expression for NKB and mRNA expression for dynorphin within the ARC of prepubertal, peripubertal, and postpubertal female ewe lambs to determine the developmental relationship of NKB and dynorphin with kisspeptin. Given their respective roles in regulating GnRH/LH secretion, we hypothesized that kisspeptin and NKB expression would increase while dynorphin mRNA expression would decrease in association with puberty-related changes in LH secretion (Figure 1).

## 2. Methods

### 2.1. Animals

Twenty-eight black faced lambs of primarily Hampshire and Suffolk breeding were purchased from a local producer at 4 months of age and used in this experiment. Animal experiments were conducted at West Virginia University’s Farm Animal Research Facility in Morgantown, West Virginia, in accordance with West Virginia Animal Care and use Committee-approved protocols and National Institutes of Health guidelines on the care and use of animals in research. Animals were housed indoors where they received a complete sheep ration (Southern States Sheep Feed Pellets, Cargill Animal Nutrition, Minneapolis, MN, USA) containing 16% protein and 2.6% fat supplemented with Timothy pellets (Greenway Animal Nutrition, Clinton, ON, Canada) and had free access to water and a sheep-specific mineral block. Ewes were housed two per pen (2.06 × 2.06 m) on raised flooring with a clear view of other sheep. As animals of the Hampshire and Suffolk breeds are sensitive to photoperiodic influences on reproduction, lighting was controlled to simulate natural changes in day length. Photoperiodic conditions for each group at the time of blood sample and tissue collection are shown in Table 1.

### 2.2. General Methods

#### 2.2.1. Surgical Procedures

Ovariectomies were performed using aseptic conditions as previously described [42]. Briefly, a mid-ventral incision was used to access the ovaries and once exteriorized, the blood supply was first cauterized, and then cut, using an electrical ligature system (Liga-A-Sure, Medtronic, Minneapolis, MN, USA). Ovaries were then removed and incisions in the peritoneum and skin closed with suture. At the time of OVX, each ewe also received a 1-cm Silastic^®^ (inner diameter 0.34 cm, outer diameter 0.45 cm; Dow Corning Corp., Midland, MI, USA) implant containing crystalline E_2_ (~45 mg per implant) placed sc in the axillary region. These implants are identical to those used previously, which constantly produce circulating concentrations of E_2_ of approximately 4 pg/mL for several months [10,47] and which produce levels of E_2_ that are similar to those produced during the estrous cycle [48]. Animals were allowed to recover two weeks before tissue collection.

#### 2.2.2. Blood and Tissue Collection

One day before euthanasia and tissue collection, blood samples were collected every 12 min by jugular venipuncture for four hours. The samples were placed in heparinized tubes and plasma was collected and stored at −20 °C until assessed for LH by radioimmunoassay. At the end of the blood sample collection period, ewes were treated with heparin (20,000 U) 10 min before and immediately before an intravenous overdose with sodium pentobarbital (Euthasol, 50 mg/kg, Webster Veterinary, Devens, MA, USA). After loss of eye reflex and cessation of breathing, the head was removed and the brain was perfused through the carotid arteries with 5 L of 4% paraformaldehyde in 0.1 M phosphate buffer (PB, pH 7.4). Blocks of tissue including the hypothalamus and preoptic area were removed and stored in paraformaldehyde for 24 h at 4 °C. Tissue was transferred to 20% sucrose in PB at 4 °C until sectioned. Frozen coronal sections of tissue were cut at a thickness of 45 μm on a freezing microtome and stored in cryopreservative until staining.

### 2.3. Determination of Age-Dependent mRNA and Protein Expression for Kisspeptin, NKB, and Dynorphin

Five groups of female lambs were used to assess changes in NKB and kisspeptin protein expression or NKB, kisspeptin, and dynorphin mRNA expression during pubertal development. Blood samples and hypothalamic tissue were collected at 5 months (*n* = 6), 6 months (*n* = 6), 7 months (*n* = 5), 8 months (*n* = 5), and 10 months (*n* = 6) of age. These ages were picked to represent the prepubertal (5–7 months), peripubertal (8 months), and postpubertal (10 months) periods of development (Table 1).

For protein expression assessment, immunohistochemistry was used to detect NKB- and kisspeptin-positive cells in the medial to caudal region of the ARC. Three hemi-sections per animal were used for each time point. On day 1 of the protocol, sections were washed 12 × 5 min in 0.1 M phosphate buffered saline (PBS) to remove excess cryopreservative. The sections were then incubated in 1% H_2_O_2_ for 10 min and then washed 3 × 5 min in PBS. Sections were subsequently incubated in a blocking solution containing PBS, 0.4% Triton-X100 (PBNT; Sigma-Aldrich, St. Louis, MO, USA), and 20% Normal Goat Serum (NGS; Jackson ImmunoResearch Laboratories, Inc., West Grove, PA, USA) for 1 h. Next, tissue sections were incubated in rabbit anti-kisspeptin (a gift from Alain Caraty, INRA #566; [49]) diluted 1:50,000 in PBNT and 20% NGS overnight. On day 2, the tissue sections were washed 3 × 5 min in PBS before incubation with biotinylated goat anti-rabbit IgG (Cat# BA-1000, Vector Labs, Burlingame, CA, USA) diluted 1:400 in PBNT and 20% NGS for 1 h. Tissue sections were washed 3 × 5 min in PBS prior to incubation in a solution containing streptavidin horseradish-peroxidase conjugate (Vectastain Elite ABC, 1:600; Vector Laboratories) for 1 h. The sections were subsequently washed in PBS and incubated in biotinyl-tyramide (Cat# NEL 700A, Perkin Elmer, Waltham, MA, USA) diluted 1:250 in PBS with 1 μL/mL of 3% H_2_O_2_ for 10 min. Tissues were washed again and covered from this point forward to protect fluorescent labeling. The tissue was afterward incubated in Dylight green conjugated to streptavidin (Cat# S11223, Fisher Scientific, Hampton, NH) 1:200 in PBS for 1 h. Tissues were washed and incubated in PBNT and 20% NGS for another hour and then incubated in rabbit anti-NKB (Cat.#H-046-26, RRID: AB_2716809; Phoenix Pharmaceuticals Inc., Burlingame, CA, USA; [50]) diluted 1:250 in PBNT and 4% NGS overnight. On day 3, tissues were washed and incubated in anti-rabbit Alexa 555 (Cat #A-21428, Fisher Scientific, Hampton, NH, USA) diluted 1:200 in PBNT and 20% NGS. The tissue was then washed, mounted on Superfrost microscope slides (Fisher Scientific), and coverslipped with Gelvatol.

For detection of mRNA for kisspeptin, NKB, and dynorphin, fluorescent in situ hybridization (RNAscope) was performed on ARC hemi-sections (2 mid-ARC hemisections/animal; 45 µm thick) using 5-, 8-, and 10-month old ewes. While the specificity of each probe yields high confidence in the results, the specificity results in limitations such as each probe being restricted to one channel. Therefore, two series of RNAscope experiments were conducted to compensate for these limitations at the time: the first series detected mRNA for kisspeptin and dynorphin, and the second series detected mRNA for NKB (see Table 2 for details on RNAscope probes). Briefly, RNAscope was performed on two medial ARC hemisections (at least 250 μm apart) per animal per series using the RNAscope Multiplex Fluorescent Reagent Kit v2 (Cat# 321710, Advanced Cell Diagnostics, Newark, CA, USA), and all incubations at 40 °C were completed using an ACD HybEZ II Hybridization System with EZ_Batch Slide System (cat#321710, Advanced Cell Diagnostics, Newark, CA, USA). For day 1, the procedure was identical between the two experimental series; hemisections were incubated in 0.1 M phosphate buffered saline (PBS; pH = 7.4) on a rocking shaker at 4 °C overnight. On day 2, which was also identical between the two series, hemisections were mounted onto Superfrost/Plus microscope slides (Fisher Scientific, Waltham, MA, USA), allowed to air dry for 2 h, and then slides were heated on a slide warmer to 60 °C for 90 min. The slides were then incubated in 4% PFA at 4 °C for 1 h, rinsed four times in 0.1 M PBS (5 min/rinse), and placed in increasing concentrations of ethanol (50%, 70%, 100%, and 100%) for 5 min per concentration. Subsequently, the slides were air-dried at room temperature (RT) for 5 min followed by incubation in H_2_O_2_ solution (Cat# 322001, Advanced Cell Diagnostics, Newark, CA, USA) for 10 min at RT. Next, slides were briefly rinsed three times with deionized water, incubated with Target Retrieval solution (Cat# 322001, Advanced Cell Diagnostics) for 10 min at 94 °C, and rinsed three times in deionized water, submerged in 100% ethanol three times, and then allowed to air dry. A hydrophobic barrier was created around each hemisection using an ImmEdge Pen (Cat# 310018, Advanced Cell Diagnostics), and slides were stored overnight at RT. On day 3, the procedure varied between the two series, as series 2 (detection of mRNA for NKB) had one less probe. Details are included to denote these differences accordingly. Sections were treated with RNAscope Protease III (Cat# 322337, Advanced Cell Diagnostics) for 30 min at 40 °C. Probes for target genes and positive controls were mixed at a concentration of 50:1:1 for the channel 1 probe, channel 2 probe, and channel 3 probe, respectively. For channels without probes (i.e., series 1—channel 1 and series 2—channels 2 and 3), an equivalent amount of RNAscope Probe Diluent (cat# 300041, Advanced Cell Diagnostics) was used to replace the corresponding volume. All probe solutions including the negative control solution were heated to 40 °C for 10 min in a water bath and cooled to RT before application. Following Protease III, tissue for series 1 was incubated with RNAscope target probes (Advanced Cell Diagnostics) for dynorphin (Cat# 481421-C2, Oa-PDYN-O1-C2) and kisspeptin (Cat# 497471-C3, Oa-KISS1-C3). Tissue for series 2 was incubated with RNAscope target probe for NKB (Cat# 481411, Oa-TAC3-O1). Control tissue was included in both series, where hemisections were incubated with respective control probes (positive controls: Cat# 516171Oa-POLR2A; Cat# 457031-C2, Oa-PPIB; Cat#516181-C3, Oa-UBC-C3; negative control: Cat# 320871, 3-plex Negative Control Probe) for 2 h at 40 °C. Next, all slides from both series were washed twice (2 min each) at RT with 1× Wash Buffer (Cat# 310091, Advanced Cell Diagnostics) followed by sequential tissue application and incubation of the following at 40 °C with 2 min washes using 1× Wash Buffer between applications: RNAscope Multiplex FL v2 AMP 1 (Cat# 323101, Advanced Cell Diagnostics) for 30 min, RNAscope Multiplex FL v2 AMP 2 (Cat# 323102, Advanced Cell Diagnostics) for 30 min, and RNAscope Multiplex FL v2 AMP 3 (Cat# 323103, Advanced Cell Diagnostics) for 15 min. Following the final incubation with AMP 3, slides were rinsed twice (2 min each) with 1× Wash Buffer at RT. To develop signal for probes in series 1 (mRNA of kisspeptin and dynorphin), hemisections received application of RNAscope Multiplex FL v2 HRP-C2 (Cat# 323106, Advanced Cell Diagnostics) for 15 min at 40 °C. Sections were next incubated with Opal 570 (Cat# NC1601878, Fisher Scientific) in RNAscope TSA buffer at a final concentration of 1:1500 for 30 min at 40 °C, followed by two rinses (2 min each) in 1× Wash Buffer at RT. Then, RNAscope Multiplex FL v2 HRP Blocker was applied to tissue for 15 min at 40 °C. Finally, slides were then rinsed twice (2 min each) with 1× Wash Buffer at RT followed by tissue application of RNAscope Multiplex FL v2 HRP-C3 (Cat# 323106, Advanced Cell Diagnostics) for 15 min at 40 °C. Sections were next incubated with Opal 520 (Cat# NC1601877, Fisher Scientific) in RNAscope TSA buffer at a final concentration of 1:1500 for 30 min at 40 °C, followed by two rinses (2 min each) in 1× Wash Buffer at RT. Then, RNAscope Multiplex FL v2 HRP Blocker was applied to tissue for 15 min at 40 °C. Finally, slides were coverslipped with Invitrogen ProLong Gold Antifade Mountant (Cat# P36930, Fisher Scientific) and stored at 4 °C until image acquisition. To develop signal for probes in series 2 (mRNA of NKB), slides received tissue application of RNAscope Multiplex FL v2 HRP C1 (Cat# 323104, Advanced Cell Diagnostics) for 15 min at 40 °C. Next, sections were incubated with Opal 690 (Fisher Scientific; cat# NC1605064) in RNAscope TSA buffer (Cat# 322809, Advanced Cell Diagnostics) at a final concentration of 1:1500 for 30 min at 40 °C. Following two rinses (2 min each) with 1× Wash Buffer at RT, RNAscope Multiplex FL v2 HRP Blocker (Cat# 323107, Advanced Cell Diagnostics) was applied to tissue for 15 min at 40 °C. Slides were then rinsed twice (2 min each) with 1× Wash Buffer at RT. Finally, slides were coverslipped with Invitrogen ProLong Gold Antifade Mountant and stored at 4 °C until image acquisition.

### 2.4. Data Analysis

#### 2.4.1. Assays

LH was assessed by radioimmunoassay as previously described [51]. Three criteria were used to identify an LH pulse; (1) a peak within two samples of the previous nadir; (2) amplitude greater than sensitivity of the LH assay; and (3) three standard deviations larger than the nadir directly preceding and following it. The limit of detection was 0.07 ng/mL with intra- and interassay coefficients of variation being 8.4 and 9.3%, respectively.

#### 2.4.2. Immunocytochemical Assessment of Kisspeptin and NKB Cell Numbers

Tissue sections were imaged using a Slide Scanner microscope (VS120 Slide Scanner; Olympus, Tokyo, Japan) wherein Z-stack images were obtained (twenty-five per cell at 2 μm each). The number of neurons containing kisspeptin and NKB were counted by an observer blinded to treatment using OlyVia software (Olyvia Ver.2.9.1; Olympus, Tokyo, Japan). The percentage of kisspeptin neurons expressing NKB and vice versa was also assessed.

#### 2.4.3. RNAscope in Situ Hybridization

Imaging of mRNA for kisspeptin and dynorphin was completed using a Zeiss 880 confocal laser scanning microscope. Imaging of mRNA for NKB was completed using a Zeiss 710 confocal laser scanning microscope. The number of cells that expressed mRNA for kisspeptin, NKB, and dynorphin was quantified by a blinded observer, with images taken from two non-overlapping confocal z-stack images, one dorsal-medial and one ventral-medial, at 1 µm optical sections of the middle ARC per hemisection using a Plan Apochromat 20×/0.8 dry objective with consistent acquisition settings for all images within their respective RNAscope series. Each image was opened using Zen 2.3 SP1 Black (Zeiss, Oberkochen, Germany), where individual cells were marked using ScreenMarker MFC Application 1.0.0.1 (Uptodown, Málaga, Spain), ensuring that each cell was only counted once. Then, the blinded observer used Fiji/ImageJ [52] to quantify the average number of cells expressing each transcript. To determine the integrated density for each neuropeptide within cells of the ARC, transcripts were examined in cells that coexpressed mRNA for kisspeptin and dynorphin (10 cells/animal) and in cells that expressed mRNA for NKB (10 cells/animal), which were randomly identified and selected for analysis. Using a Zeiss 880 confocal laser scanning microscope for all individual cells, confocal z-stack images that encompassed each cell were captured at 1 µm optical sections through the cell with a Plan Apochromat 63×/1.4 oil objective with acquisition settings held constant for all images. Following image acquisition, an observer blinded to group converted images to 8-bit using Fiji/ImageJ and applied a region of interest (312 × 312 pixels) directly over each cell to determine integrated density. An automatic minimum threshold was recorded for each channel corresponding to its specific label in all optical slices in order to calculate an average threshold for each channel. These respective averages were used as the fixed threshold intensity for integrated density analysis to normalize results across treatment. Three optical slices from the center of each cell, as determined by the extent of detectable signal throughout the cell, were used for analysis with the sum of the integrated density values calculated per cell and then averaged per animal for statistical comparison.

#### 2.4.4. Statistical Analysis

Mean LH, peptide cell numbers, and RNAscope endpoints were compared by one-way ANOVA, followed by Tukey’s post hoc analysis when a significant effect of age was detected. LH pulse frequency data were analyzed by Friedman repeated measures ANOVA. Due to a lack of normality within the data for kisspeptin mRNA integrated density per cell, data were log transformed and a one-way ANOVA on ranks was performed. Significance was declared at *p* < 0.05 and a tendency was reported at 0.05 ≤ *p* < 0.1. Statistical analyses were performed using GraphPad Prism software (GraphPad Software, San Diego, CA, USA).

## 3. Results

### 3.1. Changes in LH Secretion with Age

**Figure 2:**
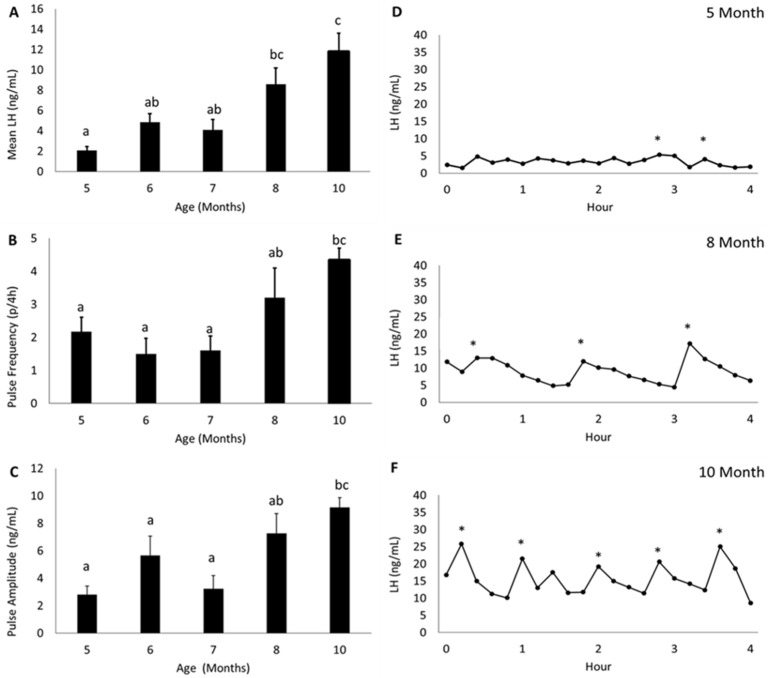
Mean LH (±SEM) (**A**), LH pulse frequency (±SEM) (**B**), and LH pulse amplitude (±SEM) (**C**) in OVX+E_2_ lambs at 5, 6, 7, 8, and 10 months of age. Mean LH, LH pulse frequency, and LH pulse amplitude were lowest during the prepubertal ages of 5, 6, and 7 months, then increased to intermediate levels at the peripubertal time period of 8 months, and then were elevated above prepubertal values at the postpubertal age of 10 months. Differing letter superscripts denote significant (*p* < 0.05) differences. Panels (**D**–**F**) showing representative LH secretory profiles for individual lambs at 5, 8, and 10 months. Peaks of LH pulses are identified by asterisks. Number of animals: 5 months (*n* = 6), 6 months (*n* = 6), 7 months (*n* = 5), 8 months (*n* = 5), 10 months (*n* = 6). * *p* < 0.05.

Mean LH, LH pulse frequency, and LH pulse amplitude followed a similar pattern of expression over time (Figure 2). Mean LH (Figure 2A), LH pulse frequency (Figure 2B) and LH pulse amplitude (Figure 2C) were significantly lower (*p* < 0.05) at five, six, and seven months of age than they were at ten months of age. Mean LH (Figure 2A), LH pulse frequency (Figure 2B), and LH pulse amplitude (Figure 2C) were intermediate at eight months of age and did not differ (*p* > 0.10) significantly from values for five, six, and seven or ten months of age. Representative patterns of LH secretion in individual animals at five, eight, and ten months of age are shown in Figure 2D–F.

### 3.2. Kisspeptin, NKB, and Dynorphin Expression

**Figure 3:**
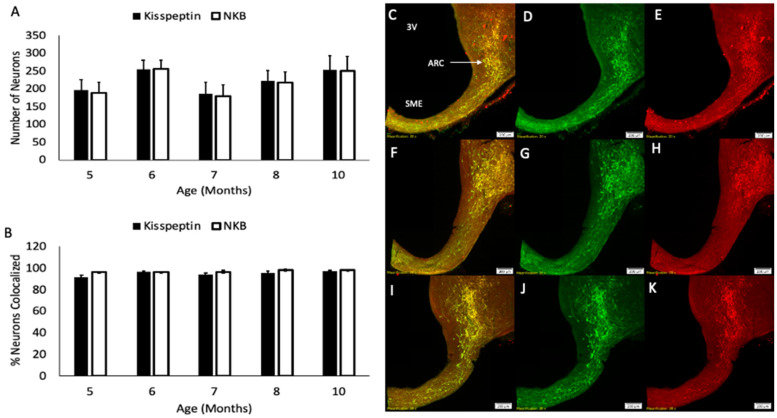
Mean (±SEM) number of kisspeptin (black bars) and NKB (open bars) neurons (**A**) in female sheep at 5, 6, 7, 8, and 10 months of age as assessed by dual-label immunofluorescence. Mean (±SEM) percentage of kisspeptin neurons coexpressing NKB (black bars) and NKB neurons coexpressing kisspeptin (open bars) in those same lambs (**B**). Representative photomicrographs of kisspeptin (green), NKB (red), and merged (yellow) immunostaining in lambs at 5 months (**C**–**E**), 8 months (**F**–**H**), and 10 months (**I**–**K**) of age. Scale bars = 200 µm. Number of animals: 5 months (*n* = 6), 6 months (*n* = 6), 7 months (*n* = 5), 8 months (*n* = 5), 10 months (*n* = 6).

Analysis of variance revealed that neither kisspeptin- nor NKB-immunopositive cell numbers varied significantly (*p* > 0.20) across age group (Figure 3A). Likewise, the percentage of kisspeptin neurons expressing NKB (overall, 95.1%) or the percentage of NKB neurons expressing kisspeptin (overall, 97.0%) did not differ (*p* > 0.10) with age (Figure 3B). Representative examples of immunostaining for kisspeptin and NKB in five-month old (prepubertal), eight-month old (peripubertal), and ten-month old (postpubertal) females are shown in Figure 3C–K. Robust immunostaining for kisspeptin and NKB can be seen at all ages. Dynorphin protein expression was not examined by immunohistochemistry as we previously showed the dynorphin expression within the ARC of prepubertal female sheep is virtually absent [42].

**Figure 4:**
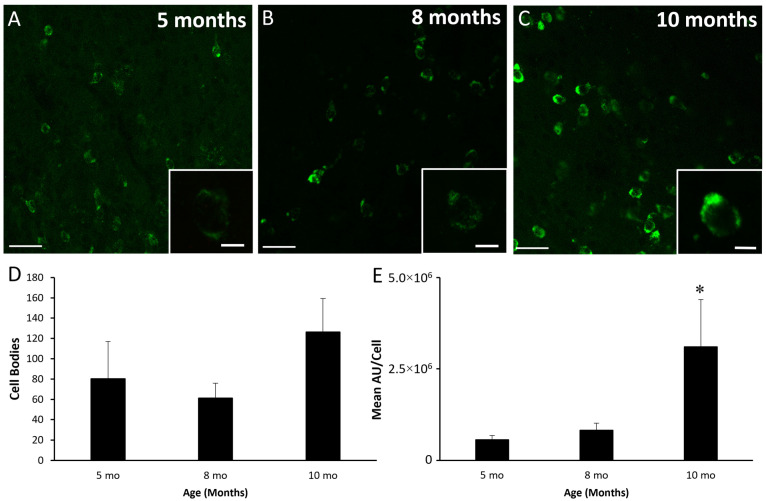
Kisspeptin mRNA (*Kiss1*) in the arcuate nucleus of female sheep at different ages. Low-power confocal images (20× objective) from a 5- (**A**), 8- (**B**), and 10-month-old female (**C)**. Insets are a single-plane representative cell taken at high-power (63× objective) within the respective section. (**D**) Mean (±SEM) number of cells expressing mRNA for kisspeptin. (**E**) Mean (±SEM) integrated density per cell. Asterisk denotes a tendency (*p* = 0.065) for the values at month 10 to be greater than those at month 5. Scale bar, 50 µm. Inset scale bar, 10 µm. Cells expressing mRNA for kisspeptin were artificially colored green. Number of animals: 5 months (*n* = 6), 8 months (*n* = 5), 10 months (*n* = 6).

**Figure 5:**
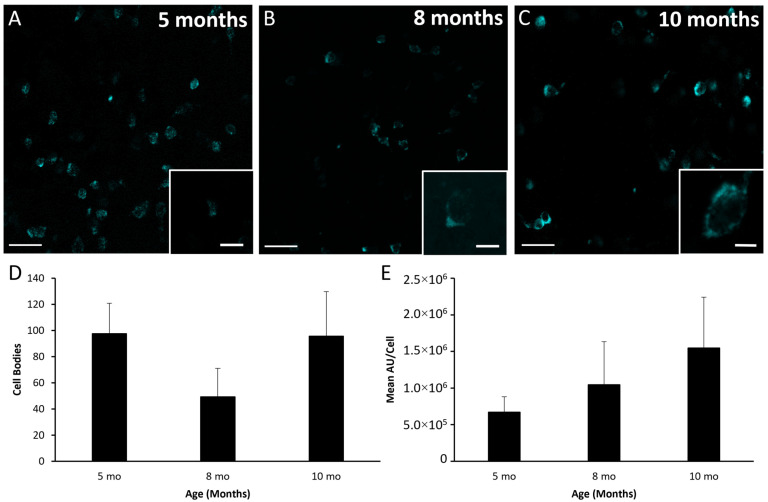
NKB mRNA (*TAC3*) in the arcuate nucleus of female sheep at different ages. Low-power confocal images (20× objective) from a 5- (**A**), 8- (**B**), and 10-month (**C**) female. Insets are a single-plane representative cell taken at high-power (63× objective) within the respective section. (**D**) Mean (±SEM) number of cells expressing mRNA for NKB mRNA. (**E**) Mean (±SEM) integrated density per cell. Scale bar, 50 µm. Inset scale bar, 10 µm. Cells expressing mRNA for NKB were artificially colored blue. Number of animals: 5 months (*n* = 6), 8 months (*n* = 5), 10 months (*n* = 6).

**Figure 6:**
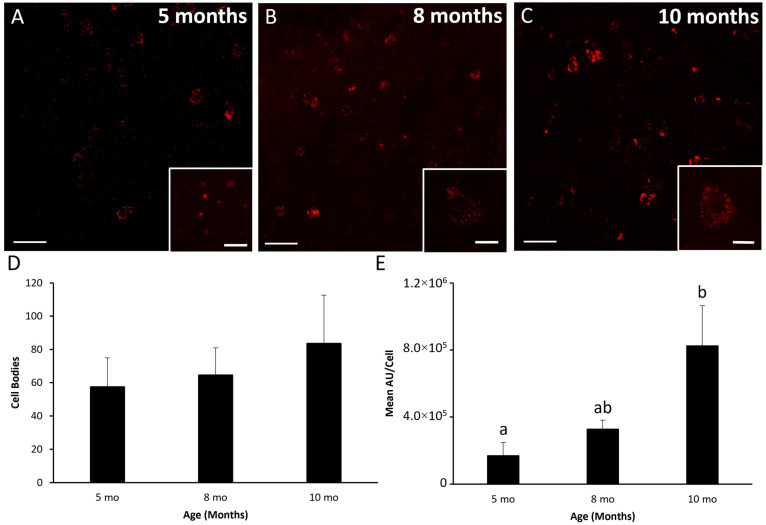
Dynorphin mRNA (*PDyn*) in the arcuate nucleus of female sheep at different ages. Low-power confocal images (20× objective) from a 5- (**A**), 8- (**B**), and 10-month (**C**) female. Insets are a single-plane representative cell taken at high-power (63× objective) within the respective section. (**D**) Mean (±SEM) number of cells expressing mRNA for dynorphin. (**E**) Mean (±SEM) integrated density per cell. Differing letter superscripts denote significant (*p* < 0.05) differences. Scale bar, 50 µm. Inset scale bar, 10 µm. Cells expressing mRNA for dynorphin were artificially colored red. Number of animals: 5 months (*n* = 6), 8 months (*n* = 5), 10 months (*n* = 6).

Data derived from RNAscope assessment of mRNA for kisspeptin, NKB, and dynorphin are shown in Figure 4, Figure 5 and Figure 6, respectively. Representative photomicrographs of ARC mRNA for kisspeptin (i.e., *Kiss1* mRNA) in five-, eight-, and ten-month-old females are shown in Figure 4A–C. The numbers of cells expressing mRNA for kisspeptin (Figure 4D) were not different (*p* > 0.30) amongst lambs at five, eight, and ten months of age. There was a tendency for the integrated density of mRNA for kisspeptin within cells to increase from five to ten months (*p* = 0.065, Figure 4E). However, there was relatively high variability at ten months of age, which was largely due to elevated levels in a single animal.

Representative photomicrographs of ARC mRNA for NKB (i.e., *TAC3* mRNA) are shown in Figure 5A–C. The number of cells expressing mRNA for NKB (Figure 5D) did not differ (*p* > 0.30) with age, nor was there any difference (*p* > 0.50) in integrated density of mRNA for NKB per neuron (Figure 5E). Representative photomicrographs of ARC mRNA for dynorphin (i.e., *PDyn* mRNA) are shown in Figure 6A–C. The number of neurons expressing mRNA for dynorphin did not differ (*p* > 0.50) with age (Figure 6D). However, integrated density of mRNA for dynorphin per cell (Figure 6E) was greater (*p* < 0.04) at ten months than at five months.

## 4. Discussion

Puberty in mammalian species is dependent upon an increase in pulsatile GnRH and LH secretion that promotes ovarian follicular development and corresponding increases in E_2_ release that trigger the GnRH/LH surge and an initial ovulation. Since this increase in pulsatile GnRH/LH release is due to an escape from E_2_-negative feedback, puberty onset can be modeled by ovariectomizing animals and replacing with constant release E_2_ implants. In our study, relatively low mean LH values and pulse frequencies characterized the prepubertal period at five, six, and seven months of age, were intermediate at the peripubertal age of eight months, and then were elevated at the postpubertal age of ten months. This provided an optimal timeline in which to examine any changes in KNDy peptide mRNA or protein expression associated with puberty-related changes in LH secretion. Consistent with a previous report detailing a lack of change in kisspeptin mRNA expression in female sheep [46], we found no significant changes in kisspeptin mRNA or protein-containing cell numbers, although we did detect a tendency for an increase in integrated density for kisspeptin mRNA at ten months of age compared to five months. As the pubertal increase in LH secretion could be due to increased NKB input or decreased dynorphin input, this study expanded upon that previous work and found that no increase in NKB-cell containing numbers or mRNA expression nor any decrease in mRNA expression for dynorphin occurred. Thus, at least in sheep, the pubertal increase in LH secretion appears to occur absent of large changes in KNDy protein or mRNA expression.

The critical nature of kisspeptin in regulating puberty onset has been demonstrated in both humans and rodents [21,22,23,24] through natural or induced deletions in the gene for the peptide or its receptor. In rodents, an early study reported increased kisspeptin mRNA expression associated with puberty onset [29], although kisspeptin mRNA in this instance was measured in whole hypothalami and, thus, changes specifically in KNDy neurons could not be determined. Subsequently, studies using mice reported an increase in kisspeptin mRNA expression relative to puberty onset [53,54,55], whereas some others reported no change [25,56]. More recently, an extensive timeline was examined in mice [57] and the authors reported that the numbers of kisspeptin mRNA-expressing neurons increased during pubertal development. Interestingly, the abundance of mRNA expression per cell peaked at postnatal day 15 and then dropped significantly by day 20 and remained unchanged through day 30, a time by which all mice had experienced vaginal opening. These authors also assessed expression of *cfos*, a marker of neuronal activation, in kisspeptin neurons and noted that no significant changes in the percentage of kisspeptin neurons expressing *cfos* occurred during pubertal development. In primates, kisspeptin mRNA expression increases during the mid-pubertal phase of development, and this process influences the increment in kisspeptin release from the median eminence as measured by push–pull perfusion [58,59]. In sheep, our previous study showed that kisspeptin-containing cell numbers were higher in ovary-intact postpubertal females during the early part of the follicular phase versus prepubertal females [40]. In the current study, we did not find a change in kisspeptin immunopositive cells numbers, which differs from our previous work. The reasons for the disparate outcomes are not completely clear, but may be due to the difference in the animal model used (OVX+E2 vs. ovary-intact) as it is possible that elevated kisspeptin cell numbers in our previous study could have occurred in anticipation of the GnRH surge [60] as animals were in the early part of the follicular phase. In the current study, we found a tendency for kisspeptin mRNA expression to increase in the lambs of a postpubertal age, raising the possibility of an increase in mRNA expression per cell that may be associated with increased GnRH/LH secretion. This is in contrast with previous findings [46], where it was reported that kisspeptin mRNA expression did not differ between OVX females implanted with E_2_ that were classified as prepubertal, peripubertal, or postpubertal based on LH secretory patterns. However, those authors did note that LH pulse frequency was positively correlated to kisspeptin cell numbers in the medial ARC, which would be in keeping with our tendency for increased kisspeptin mRNA expression in the lambs at ten months of age that exhibited the highest LH pulse frequencies. As we found no change in immunopositive kisspeptin cell numbers despite the tendency for an increase in kisspeptin mRNA expression, it is possible that changes in mRNA expression may not be faithfully reflected in protein synthesis or that changes in protein content per cell may not be detectable using an immunocytochemical approach. Regardless, we found no significant changes in kisspeptin-containing cell numbers with age using immunofluorescence in those same animals, even though expected changes in pulsatile LH secretion did occur. Indeed, kisspeptin protein expression was readily evident by five months of age, a time well before the onset of puberty in our animals. This would suggest that puberty-related changes in LH secretion occur without significant changes in kisspeptin protein or mRNA expression in female sheep.

Evidence that NKB is important in regulating LH secretion originated from early studies showing that NKB in the infundibular nucleus was elevated in postmenopausal women, who also display elevated LH secretion [61]. Subsequently, it was found that naturally occurring deletions of NKB or its receptor, NK3R, led to infertility and an absence of puberty onset in humans [34,35], although a much milder phenotype was noted in mice with NKB deletions [36,37]. In rodents, expression of mRNA for the NKB gene, *Tac2*, is elevated in a puberty-related fashion [62,63]. In the previously mentioned study by Semaan and Kauffman [57], *Tac2* expression gradually increased throughout peripubertal ages, reaching adult-like levels by postnatal day 24. *Tac2* expression per cell and total ARC expression followed a relatively similar pattern. Only extremely limited data are available regarding puberty-related changes in NKB gene expression in primates or humans. Taziaux and coworkers [64] reported that the volume of NKB immunoreactivity or numbers of NKB-immunopositive neurons in the infundibular (a.k.a. ARC) nucleus from postmortem tissue of infantile/pubertal human females did not differ from that of adult females. However, the ages in the former group were limited in number and ranged from five months to 13 years in age, so any puberty-specific changes could not be ascertained. Much like kisspeptin protein expression, we saw no age-related changes in NKB protein expression. In our previous study [40], we reported that NKB-containing cell numbers did not differ between ovary intact prepubertal females and postpubertal females in the early part of the follicular phase, but increased following ovariectomy in both groups. Given the increase in LH that occurred in the face of constant E_2_ concentrations in our current study, indicative of an escape from E_2_-negative feedback, we would have predicted that NKB cell numbers or mRNA abundance would have increased, but we found no such age-related changes. It is unclear as to why this was the case, but we did see greater fiber staining density in prepubertal versus postpubertal ewes in our previous work [40], raising the possibility of greater synthesis, transport, and release that may not be reflected by an assessment of cell numbers. Increased NKB synthesis would be consistent with the numerical increase in integrated density of expression per cell for NKB mRNA from five to eight to ten months of age, but these increases were not statistically significant. A lack of significant change in NKB mRNA expression is consistent with a previous study in sheep that reported no change in total cell numbers for NKB or the amount of mRNA for NKB per cell amongst ovary-intact prepubertal female sheep, postpubertal female sheep in the luteal phase of the estrous cycle, and postpubertal sheep that had been ovariectomized for one week [65].

The role of dynorphin in puberty has been much less studied than that of kisspeptin and NKB. In mice, administration of nor-BNI, a kappa-opiate receptor antagonist, accelerates puberty onset and is associated with an increase in LH secretion [66]. Our previous work [42] showed that central administration of nor-BNI increased pulsatile LH secretion in female sheep of a prepubertal age that were OVX and implanted with E_2_. A similar treatment in those same sheep at a postpubertal age was without effect. These studies strongly suggest that dynorphin may be part of the “prepubertal brake” that inhibits GnRH secretion prior to puberty. Interestingly, in our previous study [42], we were unable to detect dynorphin via immunocytochemistry in prepubertal lambs even though it was abundant in tissue from adult ewes in the luteal phase of the estrous cycle that was run concurrently, as also seems to be the case in young female pigs (Nestor, unpublished data). Thus, in the current study, we examined dynorphin mRNA expression, but not protein expression. We failed to find significant age-related changes in dynorphin mRNA-expressing cell numbers within the ARC, although we noted a significant increase in dynorphin mRNA expression per cell in OVX+E_2_ lambs at a postpubertal age. The meaning of this increase isn’t quite clear since, as mentioned above, dynorphin is inhibitory to GnRH release and LH pulse frequency was elevated at this age. It may be that the increase in integrated density of mRNA for dynorphin within the ARC may reflect some type of intracellular feedback loop where decreased levels of peptide may result in increased mRNA expression. An increase in mRNA for dynorphin is consistent with the previous work of Li et al. [65], wherein dynorphin mRNA expression was higher in postpubertal than prepubertal ewes. However, this result should be viewed with caution as that study compared prepubertal ovary-intact ewes to postpubertal ewes in the luteal phase of the estrous cycle and progesterone has been shown to increase dynorphin in female sheep [67,68]. Although an increase in ARC dynorphin does not support our hypothesis, it is consistent with the possibility raised in our previous work that ARC dynorphin may not be the source of inhibition noted to occur prior to puberty in female sheep and thus any alterations in peptide or mRNA expression within the ARC would be disconnected from changes in GnRH/LH secretion. In addition, as GnRH neurons also express kappa-opiate receptors [42], effects of dynorphin on GnRH release may occur directly or within locations outside of the ARC. To date, we have been unable to elicit an increase in LH secretion following implantation of nor-BNI within either the preoptic area or ARC (unpublished data). Thus, the source of prepubertal inhibition by dynorphin and where that inhibition is exerted remains to be determined.

The increase in pulsatile GnRH/LH release that heralds puberty onset in domestic livestock species and rodents species involves an escape from the negative feedback actions of estradiol [9]. Although in primates, the peripubertal increase in GnRH and LH is steroid-independent, the period between menarche and establishment of consistent menstrual cycles is characterized by the imposition of estrogen negative feedback [13]. As GnRH neurons do not express ERa, the isoform necessary for regulating GnRH secretion, it is believed that inhibitory effects of estrogen are exerted through other neurons afferent to GnRH neurons. Given their key role in regulating fertility and that the vast majority express ERa, KNDy neurons are well-situated to play that role. However, we previously concluded that changes in the expression of ERa, particularly in kisspeptin neurons in the ARC, do not explain the pubertal escape from E_2_-negative feedback in ewe lambs [69]. The fact that we found abundant kisspeptin and NKB cell numbers well before puberty would normally occur and that immunopositive cell numbers did not change with age may suggest that, while critical for reproduction, a reduction in E_2_-negative feedback at the level of KNDy neurons or escalation of kisspeptin or NKB protein expression is not the trigger for puberty onset. This is supported by the finding that although there was a gradual increase in kisspeptin and NKB mRNA-containing cell numbers in mice with puberty, cfos expression in those neurons did not change [57], suggesting that an increase in activation of those neurons did not occur with puberty. That KNDy neurons are not the actual trigger for puberty onset in primates was also suggested by Garcia et al. [70] who postulated that the increase in kisspeptin release associated with puberty was dependent upon an escape from a not-as-yet completely characterized inhibitory input. As KNDy neurons are believed to represent a critical component of pulsatile GnRH release, i.e., the GnRH pulse generator [45], they are integral to effecting changes in GnRH/LH secretion. However, they may not be, in and of themselves, direct targets for signals that regulate puberty onset, for instance peripheral metabolic signals such as leptin. Thus, these inputs may be transduced through other neurons such as proopiomelanocortin or agouti-related peptide/neuropeptide Y neurons that are afferent to KNDy neurons and may impact the timing of puberty in that manner. Another interesting possibility examined in our study was that changes in one KNDy peptide may precede that of the others. For example, NKB might increase early in development, but kisspeptin may not increase until later, making kisspeptin limiting to the process. However, we did not find any changes in the numbers of kisspeptin- or NKB-expressing neurons with age or the percentage of coexpression of NKB and kisspeptin, suggesting that kisspeptin and NKB develop well before puberty onset and do so along a similar timeline and, as such, expression of one does not limit the other.

## 5. Conclusions

In this study, we found that protein and mRNA expression of KNDy neuron peptides was readily evident well before the time of puberty onset in female sheep. Furthermore, outside of a statistical tendency for kisspeptin mRNA expression per cell to increase, we found no predictable changes in protein or mRNA expression that was associated with the pubertal increase in pulsatile GnRH/LH release. This may suggest that, while critical for puberty onset, the active involvement of KNDy neurons may await direction from other neurons mediating inputs arising from cues such as nutrition, photoperiod, or stress. The impact of these inputs on GnRH/LH secretion in sheep have been demonstrated [9,71], though the neural pathways mediating these signals and their relationship to KNDy neurons in this species have not been completely worked out. Future work will be necessary to establish the neurocircuitry mediating such inputs on puberty.

## Figures and Tables

**Table 1:** Age of females, the neuroendocrine state of pubertal development relative to LH secretion, the number of animals per age group, and the photoperiod conditions under which blood and tissue samples were collected for each group.

**Age**	5 months	6 months	7 months	8 months	10 months
**Neuroendocrine** **State**	Prepubertal	Prepubertal	Prepubertal	Peripubertal	Postpubertal
**N**	6	6	5	5	6
**Photoperiod**	14.5L:9.5D	13.5L:10.5D	12L:12D	11L:13D	9.5L:14.5D

**Table 2:** RNAscope probe information.

RNAscope Probe Information				
Gene Product	Probe ID	Catalog #	Accession Number	Target Region
Series 1 Probes				
Target Probe Channel 2: Dynorphin (*PDYN*)	Oa-PDYN-O1-C2	481421-C2	NM_001280677.1	2–545
Target Probe Channel 3: Kisspeptin (*KISS1*)	Oa-KISS1-C3	497471-C3	NM_001306104.1	37–774
Series 2 Probes				
Target Probe Channel 1: Neurokinin B (*TAC3*)	Oa-TAC3	481411	AJ507210.1	2–311
Positive Control Probes				
Positive Control Channel 1: Polymerase (RNA) II Subunit A	Oa-POLR2A	516171	XM_004013289.3	1197–2081
Positive Control Channel 2: Cyclophilin B	Oa-PPIB	457031-C2	XM_004010536.2	4–913
Positive Control Channel 3: Ubiquitin C	Oa-UBC-C3	516181-C3	XM_012097675.2	56–1295
Negative Control Probes				
	3-plex Negative Control Probe	320871	EF191515	414–862

## Data Availability

All data are available from corresponding author upon reasonable request.
